# Transition metal ions at the crossroads of mucosal immunity and microbial pathogenesis

**DOI:** 10.3389/fcimb.2014.00002

**Published:** 2014-01-24

**Authors:** Vladimir E. Diaz-Ochoa, Stefan Jellbauer, Suzi Klaus, Manuela Raffatellu

**Affiliations:** ^1^Department of Microbiology and Molecular Genetics, University of California, IrvineIrvine, CA, USA; ^2^Institute for Immunology, University of California, IrvineIrvine, CA, USA

**Keywords:** infection, nutritional immunity, iron, zinc, manganese, lipocalin-2, calprotectin, S100 proteins

## Abstract

Transition metal ions are essential micronutrients for all living organisms. In mammals, these ions are often protein-bound and sequestered within cells, limiting their availability to microbes. Moreover, in response to infection, mammalian hosts further reduce the availability of metal nutrients by activating epithelial cells and recruiting neutrophils, both of which release metal-binding proteins with antimicrobial function. Microorganisms, in turn, have evolved sophisticated systems to overcome these limitations and acquire the metal ions essential for their growth. Here we review some of the mechanisms employed by the host and by pathogenic microorganisms to compete for transition metal ions, with a discussion of how evading “nutritional immunity” benefits pathogens. Furthermore, we provide new insights on the mechanisms of host-microbe competition for metal ions in the mucosa, particularly in the inflamed gut.

## Introduction

Transition metal ions are involved in many biological processes crucial for sustaining life. These metals can serve as cofactors in proteins, enabling their biological function, regulating their activity, and/or stabilizing their structure (Aisen et al., 2001; Andreini et al., 2008; Waldron et al., 2009). This mini-review focuses on three metal ions targeted by host sequestration strategies and the means by which microbes acquire them; namely, iron, zinc, and manganese.

Among transition metal ions, iron is the most abundant in the human body. Of the 3–5 g of iron in adults, 65–75% is located within erythrocytes bound to heme, the tetrapyrrole cofactor of hemoglobin, and utilized for oxygen transport (Andrews, 2000). Iron is also critical for other cellular processes in mammals, such as nucleic acid and protein synthesis, electron transport, and cellular respiration (Griffiths, 1999; Lieu et al., 2001). Most of the iron in the body is intracellular, and extracellular iron is associated with high-affinity iron binding proteins, namely transferrin and lactoferrin, so iron that microorganisms need for survival is severely restricted. Much like eukaryotic cells, microorganisms also utilize iron in DNA synthesis, electron transport, oxygen binding, and superoxide metabolism (Griffiths, 1999). Outside the body, the bioavailability of iron is generally limited due to the low solubility of ferric iron (Fe^3+^) at physiological pH (7.4), likely facilitating microbial adaptation to low iron conditions (Raymond et al., 2005). In light of this, microbes that possess multiple iron uptake mechanisms, or those that can utilize alternative metal ions like zinc and manganese, are able to thrive when usable iron is scarce.

Zinc is an essential metal nutrient with an estimated dietary requirement in humans of 15 mg per day (Tapiero and Tew, 2003; King, 2011). Approximately 95% of zinc in humans is intracellular, where it serves structural and functional roles for a large number of macromolecules and enzymes (Tapiero and Tew, 2003; King, 2011). For prokaryotes, it is estimated that 5–6% of their proteome may consist of zinc binding proteins, which emphasizes the need for mechanisms of zinc acquisition in these cells (Andreini et al., 2006). In contrast to iron and zinc, only trace concentrations of manganese are found in human serum (<10 nM) and tissue (<4 μ M) (Keen et al., 2000), which is likely to pose significant challenges for microorganisms that have adapted to thrive on earth's biosphere where manganese is widely available (Morgan, 2000). Only a handful of strictly manganese-dependent enzymes are known in both eukaryotes and prokaryotes because manganese in metalloenzymes appears to be readily interchangeable with other divalent cations (Andreini et al., 2008). Manganese in microbes is largely known for its role as a cofactor for some free radical detoxifying enzymes, but it also plays a key role in central carbon metabolism (Kehres and Maguire, 2003).

Transition metal ions are important biological catalysts because they can undergo changes in oxidation states involving one electron. To limit the unspecific reactive potential of transition metals, their availability in vertebrate hosts needs to be tightly regulated at all times and especially limited during infection, a process termed nutritional immunity (the sequestration of nutrients from pathogens). Host mechanisms of nutritional immunity are varied and include: the induction of hepcidin, a master hormone regulator that controls the levels of iron in the body (Drakesmith and Prentice, 2012); the expression of the Natural Resistance-Associated Macrophage Protein 1 (NRAMP1), an ion transporter that pumps iron and manganese out of pathogen-containing phagosomes (Jabado et al., 2000; Forbes, 2003; Cellier et al., 2007); and the expression of antimicrobial proteins that sequester metal ions at sites of infection (Aujla et al., 2008; Corbin et al., 2008; Raffatellu et al., 2009; Hood et al., 2012; Liu et al., 2012). Whereas all of these strategies aid the host in limiting the replication of infecting microbes, some microorganisms have evolved or acquired mechanisms of metal uptake that circumvent the nutritional immune response. Here we review some of the mechanisms that the mammalian host utilizes to sequester metal ions in response to infection, we describe how microbes can evade this nutritional immunity (with a focus on mucosal sites), and we discuss how circumventing this host defense benefits pathogens. Although transition metal toxicity and active intoxication are also established strategies in antimicrobial host responses, we will set our focus on the starvation of essential metal nutrients.

## Microbial mechanisms of acquiring iron

Iron is required by numerous microbial species because it serves as a cofactor for important cellular processes including DNA replication, central metabolism and respiration. Microbes have thus evolved or acquired a variety of specialized iron uptake systems to overcome iron limitation. These systems are generally categorized as unbound iron, siderophore, or heme acquisition systems. Bacteria can uptake unbound iron using ferrous iron (Fe^2+^) transport systems like Feo proteins, mechanisms that appear to be important mainly during low oxygen conditions, when ferrous iron remains more stable and predominate over ferric iron (Andrews et al., 2003). Such systems likely play a negligible role in bacterial iron acquisition under inflammatory conditions, where unbound iron is rarely found.

Under iron-limiting conditions, many pathogenic bacteria and some fungi synthesize and secrete siderophores; small, high-affinity iron-chelating compounds (Neilands, 1995). Siderophore effectiveness resides in their ability to bind ferric iron (Fe^3+^) with an affinity that can exceed that of host Fe^3+^-binding proteins like transferrin or lactoferrin (Griffiths, 1999), enabling siderophores to “steal” iron from these host proteins. Microbial uptake of Fe^3+^ from siderophore-Fe^3+^ complexes is achieved by either the reduction of iron from the siderophore at the extracellular surface or by the internalization of the complex (Miethke and Marahiel, 2007). Filamentous fungi are capable of iron uptake by both routes (Philpott, 2006). Though the mechanisms of extracellular reduction by bacteria are not well-understood, the internalization of siderophore-Fe^3+^ complexes is well-studied (Crosa and Walsh, 2002; Krewulak and Vogel, 2008; Braun and Hantke, 2011). In Gram-negative bacteria, several outer-membrane receptors that transport siderophore-Fe^3+^ complexes have been identified; examples include the FepA receptor for enterobactin, and the FhuA receptor for ferrichrome (Chakraborty et al., 2007; Braun, 2009). The energy required by these receptors for the transport of the substrate originates from the proton motive force of the inner membrane and is transduced through the TonB protein complex (Braun and Braun, 2002; Moeck and Coulton, 2002; Postle and Kadner, 2003). Once in the periplasm, substrate-binding proteins (SBPs) shuttle the siderophore-Fe^3+^ complex to the corresponding ATP-binding cassette (ABC) transporter, which then translocates the complex into the cytoplasm (Biemans-Oldehinkel et al., 2006). ABC transporters in the cytoplasmic membrane of Gram-positive bacteria are also involved in the uptake of siderophore-Fe^3+^ complexes. Unlike Gram-negative bacteria, their cognate SBPs are responsible for initial binding of the complex and are tethered to the cytoplasmic membrane (Sutcliffe and Russell, 1995; Biemans-Oldehinkel et al., 2006). Once in the cytoplasm, iron can be liberated from siderophores through reduction of Fe^3+^ to Fe^2+^ or by enzymatic degradation of the siderophore (Miethke and Marahiel, 2007). Among bacteria, siderophore-based iron acquisition systems are widespread.

One of the most studied siderophores is enterobactin, also called enterochelin, which is synthesized by commensal and pathogenic Enterobacteriaceae including *Escherichia coli, Klebsiella pneumoniae*, and *Salmonella* spp (O'Brien and Gibson, 1970; Pollack and Neilands, 1970; Rogers et al., 1977; Perry and San Clemente, 1979; Lawlor and Payne, 1984). Enterobactin has high affinity for iron (*K*_*a*_ = 10^51^ M^−1^), which is higher than the affinity of host proteins like transferrin (*K*_*a*_ = 10^20^ M^−1^) (Aisen et al., 1978; Carrano and Raymond, 1979). Therefore, bacteria that synthesize enterobactin can efficiently scavenge iron from the host; however, the host innate immune response has evolved a mechanism to counteract enterobactin-mediated iron acquisition (discussed in detail below) (Fischbach et al., 2006). Although siderophores are generally secreted into the host extracellular environment, some siderophores aid iron acquisition by pathogens with a predominantly intracellular lifestyle. *Mycobacterium tuberculosis* (Mtb), for example, expresses siderophores known as mycobactins that diffuse out of Mtb-containing phagosomes, chelate iron from cytoplasmic stores, and re-enter the phagosome via lipid droplets (Luo et al., 2005).

In addition to siderophores, microbial pathogens can utilize different uptake systems to obtain iron from a variety of sources, which allow them to inhabit diverse niches and to respond to host mechanisms of iron sequestration. In the case of *Candida albicans*, uptake of unbound iron via the high-affinity iron permease *FTR1* is critical for establishing systemic infection in mice (Ramanan and Wang, 2000). In contrast, uptake of iron-bound siderophores is necessary for *C. albicans* colonization of epithelial layers but not for the development of a bloodborne infection (Heymann et al., 2002). Because *C*. *albicans* lacks the genes for the biosynthesis of siderophores (Haas, 2003), it depends on other microorganisms for the production of siderophores. Therefore, *C. albicans* uptake of iron via siderophores is likely restricted to sites where siderophore-producing microorganisms are found (e.g., mucosal surfaces in the gut). Another source of iron for microbes is the biggest pool of iron in the human body: iron from heme and heme-binding proteins.

Similar to the uptake of siderophore-bound iron, the first step in bacterial heme transport involves the binding of heme or hemoglobin to a surface receptor. In Gram-negative bacteria, TonB-dependent receptors are involved in the transport of heme into the periplasm, where heme-specific SBPs bind the molecule (Braun and Hantke, 2011). For hemoglobin, both Gram-negative and Gram-positive bacteria extract the heme group prior to transfer to an SBP. Heme-specific ABC transporters then translocate heme into the cytoplasm, where iron is released by heme-degrading enzymes (Braun and Hantke, 2011; Nobles and Maresso, 2011). Heme oxygenases catalyze the oxidative cleavage of heme with an electron donor to liberate iron (Nobles and Maresso, 2011). Subsequent catabolism of the heme is required to reduce the toxicity associated with the heme porphyrin (Nobles and Maresso, 2011).

## Host mechanisms of sequestering iron

Iron is essential for the replication of many pathogenic organisms, so it is not surprising that the host has evolved sophisticated strategies to limit the availability of iron to pathogens. Conversely, both iron supplementation and diseases characterized by iron overload, such as hemochromatosis, increase the host's susceptibility to infection (reviewed in Griffiths, 1999). In humans, the levels of unbound iron are low; most iron is bound by heme in the context of hemoglobin. Moreover, free heme can be captured by hemopexin and free hemoglobin by haptoglobin. Other proteins, like transferrin in serum, or lactoferrin in neutrophils and human secretions, bind strongly to ferric iron. In most cells, ferritin is responsible for storing iron for normal cellular use, but in specialized cells, i.e., hepatocytes and macrophages, ferritin is used for long-term iron storage and sequestration during iron overload, respectively (Andrews, 2000). Additionally, macrophages increase iron uptake and ferritin synthesis when converting to their inflammatory phenotype, suggesting that ferritin-based sequestration may be a key mechanism for intracellular iron withholding during infection (Birgegård and Caro, 2009).

An additional mechanism of regulating iron metabolism is mediated by the hormone hepcidin, which controls host-protective responses by integrating signals from iron status and threat of infection. Initially identified as an antimicrobial peptide (Krause et al., 2000), hepcidin is considered to be the master hormonal regulator of iron metabolism, controlling both the overall level of iron and its localization (Nicolas et al., 2001; Park et al., 2001; Nicolas, 2002; Nemeth et al., 2003). Upon microbial infection, the upregulation of hepcidin, concomitant with a reduction of serum transferrin saturation, causes an overall decrease in iron levels (Nemeth et al., 2003; Armitage et al., 2011). Hepcidin upregulation is partially mediated through expression of pro-inflammatory cytokines like interleukin (IL-) 6, which stimulates the production of hepcidin in the liver (Nemeth et al., 2003, 2004a; Rodriguez et al., 2013). Hepcidin then inhibits both cellular iron efflux and duodenal iron absorption by binding to and inducing the degradation of the cellular iron transporter ferroportin 1, which exports iron into the plasma from cells that store or transport iron, including hepatocytes, macrophages, and absorptive enterocytes (Nemeth et al., 2004b; Ross et al., 2012). Subcutaneous infection with either Gram-negative or Gram-positive bacteria has been shown to induce hepcidin synthesis by neutrophils and macrophages, suggesting that local production of hepcidin may limit iron availability at sites of infection (Peyssonnaux et al., 2006). Overall, the induction of hepcidin upon infection results in hypoferremia and anemia of inflammation, which represent important host defense strategies to limit the availability of iron to pathogens.

One of the most studied host transporters in the context of bacterial pathogenesis is NRAMP1,—a proton-dependent transporter of divalent metal ions expressed by professional phagocytes, such as macrophages and neutrophils (Cellier et al., 2007). This transporter is localized in the phagosomal membrane and exports Fe^2+^ and Mn^2+^ out of the phagosomal compartment, presumably to reduce access to these metals of pathogens residing within the phagosome (Cellier et al., 2007). While this export function occurs during infection, NRAMP1 is also known to contribute to hemoglobin iron recycling by reticuloendothelial macrophages that phagocytose senescent erythrocytes (Cellier et al., 2007; Soe-Lin et al., 2009). In addition to its phagosome metal-withholding function, expression of a functional NRAMP1 also restricts microbial growth by enhancing macrophage production of the antimicrobial effector molecule nitric oxide (NO) through sustained transcription of inducible nitric oxide synthase (iNOS) (Fritsche et al., 2003). Although the mechanism for iNOS induction is not fully understood, both STAT-1-mediated expression of the transcription factor IRF-1, as well as suppressed production of the inhibitory cytokine IL-10, contribute to NRAMP1-dependent prolonged activation of iNOS transcription (Fritsche et al., 2003, 2008). Similarly, another recent study using macrophage cell lines suggests that NRAMP1-mediated stimulation of the expression of lipocalin-2, an antimicrobial peptide that binds iron-loaded bacterial siderophores including enterobactin, is a novel mechanism by which NRAMP1 confers resistance to infection with the intracellular pathogen *Salmonella enterica* serovar Typhimurium (*S. Typhimurium*) (Fritsche et al., 2012). The importance of NRAMP1 in the host response to infection is further underlined by many studies showing that mice with a functional *Nramp1* (*Slc11a1*) allele are more resistant to infection with a variety of intracellular pathogens including *Mycobacterium bovis* BCG, *Leishmania donovanii*, and *S. Typhimurium* (Forbes and Gros, 2001; Cellier et al., 2007).

Host mechanisms discussed thus far effectively reduce available iron, but they are not sufficient to completely prohibit bacterial iron acquisition during an infection. As discussed above, pathogenic bacteria can deploy an efficient weapon in the battle for iron: siderophores. However, as mammals have evolved for millions of years together with siderophore-producing bacteria, it is not surprising that we have evolved an anti-siderophore mechanism: secretion of lipocalin-2 (also known as siderocalin, neutrophil gelatinase-associated lipocalin, uterocalin, or 24p3) (Goetz et al., 2002; Flo et al., 2004; Correnti and Strong, 2012). Lipocalin-2 is one of the most abundant antimicrobial proteins released by epithelial cells and neutrophils during infections in the gut and respiratory mucosa with pathogens like *S. Typhimurium* and *K. pneumoniae*, respectively (Aujla et al., 2008; Bachman et al., 2009; Raffatellu et al., 2009). Lipocalin-2 sequesters a subset of catecholate siderophores, including enterobactin, thereby limiting bacterial access to iron (Goetz et al., 2002; Flo et al., 2004; Berger et al., 2006). In a sepsis model, lipocalin-2 induction is dependent on Toll-like receptor 4 signaling (Flo et al., 2004; Srinivasan et al., 2012). It is also known that lung and intestinal epithelial cells express and secrete lipocalin-2 in response to signaling by pro-inflammatory cytokines released by T helper 17 (Th17) cells, like interleukin IL-17 and IL-22 (Aujla et al., 2008; Raffatellu et al., 2009).

In addition to lipocalin-2, IL-17 and IL-22 also stimulate epithelial secretion of neutrophil chemoattractants, known as CXC chemokines, which mediate the recruitment of neutrophils to the mucosa (Awane et al., 1999; Andoh et al., 2005; Kao et al., 2005; McAllister et al., 2005; Aujla et al., 2008; Raffatellu et al., 2009). Neutrophils play a key role in nutritional immunity because they constitute the largest proportion of circulating white blood cells in humans, quickly mobilize to sites of infection, and express high levels of antimicrobial proteins that sequester metal ions, including lipocalin-2, lactoferrin, and, as detailed below, calprotectin (Masson et al., 1969; Steinbakk et al., 1990; Goetz et al., 2002). Thus, the coordinated expression and release of metal-binding antimicrobial proteins by epithelial cells and neutrophils during infection promotes host sequestration of essential metal nutrients.

In this tug of war for iron, pathogens have evolved mechanisms to counteract the sequestration of siderophores. To circumvent this arm of nutritional immunity, pathogens including *Salmonella* species, *Klebsiella* species and uropathogenic *E. coli* (UPEC) species synthesize salmochelin, a C-glucosylated derivative of enterobactin (Hantke et al., 2003; Bachman et al., 2011), which lipocalin-2 cannot bind, thus enabling iron uptake in these species and enhancing their colonization of host tissues (Fischbach et al., 2006; Crouch et al., 2008; Raffatellu et al., 2009; Bachman et al., 2011). Evasion of lipocalin-2-mediated iron sequestration is thus regarded as a virulence mechanism. However, work in our laboratory has recently shown that a probiotic strain of the Enterobacteriaceae family (*E. coli* Nissle 1917) also evades iron sequestration by lipocalin-2 in the inflamed gut via secretion of siderophores including salmochelin (Deriu et al., 2013). In this case, iron acquisition and evasion of lipocalin-2 is beneficial to the host, because *E. coli* Nissle 1917 reduces *S. Typhimurium* intestinal colonization by outcompeting it for iron acquisition (Deriu et al., 2013). Therefore, evasion of lipocalin-2 by the secretion of modified siderophores can confer a fitness advantage to probiotic strains like *E. coli* Nissle 1917 and enhance the host response against bacterial pathogens by further sequestering iron.

## Microbial mechanisms of acquiring zinc and manganese

While the role of iron in cellular processes is well-characterized, increasing evidence suggests that other transition metal ions such as zinc and manganese also play a crucial role in microbial physiology (Keen et al., 2000; Hantke, 2005). For example, in order to circumvent host-mediated iron sequestration, *Borrelia burgdorferi* lacks most genes that code for iron-binding proteins, and, for the few metalloproteins it does express, *B. burgdorferi* uses manganese instead of iron (Posey and Gherardini, 2000). In many bacterial species, manganese also serves as a metal cofactor for proteins involved in central carbon metabolism and for the detoxification of reactive oxygen species (ROS) (Kehres and Maguire, 2003). Zinc-dependent enzymes that can detoxify ROS have also been identified (Battistoni, 2003). Furthermore, zinc was found to be associated with up to 5% of all bacterial proteins, of which more than 80% are enzymes (Andreini et al., 2006). In line with their essential role in many bacterial functions, acquisition of zinc and manganese has subsequently been shown to contribute to bacterial pathogenesis (reviewed in Kehl-Fie and Skaar, 2010).

Similar to siderophore and heme transport across the cytoplasmic membrane, ABC-type transporters are involved in bacterial uptake of zinc (Zn^2+^) and manganese (Mn^2+^) ions (Claverys, 2001). These transporter systems are composed of a cation binding protein that shuttles its substrate to its cognate transporter, a cytoplasmic ATP-binding protein that facilitates active transport, and the transmembrane protein that mediates transport through the cytoplasmic membrane. In Gram-negative bacteria, the cation binding protein is soluble and localized to the periplasm, while in Gram-positive bacteria it is a lipoprotein anchored to the extracellular membrane (Gilson et al., 1988; Tam and Saier, 1993; Sutcliffe and Russell, 1995). High-affinity ABC-type zinc transporters include ZnuABC of Gram-negative bacteria (Patzer and Hantke, 1998; Hantke, 2005), and AdcBCA of the Gram-positive streptococci (Dintilhac et al., 1997; Panina et al., 2003). ABC-type manganese transporters have also been identified in several Gram-positive and Gram-negative bacteria (Claverys, 2001; Papp-Wallace and Maguire, 2006). In *S. Typhimurium*, for example, the ABC-type transporter SitABCD is found within a pathogenicity island and is not present in the closely related organism *E. coli*, indicating this transporter could have been acquired by horizontal gene transfer (Zhou et al., 1999). Of note, studies have shown some manganese transporters to facilitate the uptake of other divalent cations such as Zn^2+^, Cd^2+^, and Fe^2+^ at lower affinities, with a *K*_*d*_ in the μM range (Kolenbrander et al., 1998; Kehres et al., 2002).

In addition to ABC-type transporters, bacteria also express homologs of the eukaryotic NRAMP transporter family (Kehres et al., 2000; Makui et al., 2000; Que and Helmann, 2000; Horsburgh et al., 2002). One example is the MntH protein of *Salmonella* and *Escherichia*, a membrane-bound, proton-coupled symporter with high specificity for manganese (Kehres et al., 2000); similar to the ABC-type manganese transporters, MntH can also transport other divalent cations at higher concentrations (Papp-Wallace and Maguire, 2006). Another discrete transporter of zinc and manganese uptake is ZupT, a permease with broad cation specificity belonging to the ZIP protein family (Grass et al., 2005; Karlinsey et al., 2010). Though metal uptake via ZupT is less specific than via the high-affinity ABC-type or NRAMP transporters, studies in *E*. *coli* have demonstrated this transporter to prefer zinc over manganese, copper, and iron (Grass et al., 2002, 2005; Taudte and Grass, 2010).

In addition to the role of these metals in essential cellular functions, evidence is mounting that specialized mechanisms of zinc and manganese acquisition contribute to bacterial pathogenesis; for instance, zinc and manganese are important cofactors in neutralizing reactive oxygen and nitrogen species, suggesting an important role for these metals in resisting these types of host antimicrobial responses (Lynch and Kuramitsu, 2000; Bowman et al., 2011). Supporting this, mutant strains of the pathogens *Brucella abortus, Pasteurella multocida*, and *S. Typhimurium* that lack the ZnuABC transporter are attenuated in systemic models of disease in mice (Campoy et al., 2002; Garrido et al., 2003; Kim et al., 2004; Ammendola et al., 2007). Furthermore, the expression of zinc transporters promotes *Campylobacter jejuni, S. Typhimurium*, and *Acinetobacter baumannii* colonization of mucosal tissues (Davis et al., 2009; Hood et al., 2012; Liu et al., 2012). For manganese acquisition, both the ABC-type transporters and the bacterial NRAMP homologs are known to contribute to systemic *S. aureus* and *S. Typhimurium* infection (Karlinsey et al., 2010; Kehl-Fie et al., 2013). Taken together, these studies indicate an important role for zinc and manganese sequestration by the host in controlling microbial infections with different pathogens.

## Host mechanisms of sequestering zinc and manganese

Compared to iron, less is known about the mechanisms the host employs to limit microbial access to metal micronutrients like zinc and manganese. Nevertheless, multiple strategies to limit the availability of these nutrients to pathogens have been identified in the mammalian host.

As described in the section on host iron sequestration, NRAMP1 is a proton-dependent exporter of Fe^2+^ and Mn^2+^ across the phagosomal membrane of vertebrates that confers resistance to various intracellular pathogens (Cellier et al., 2007). Another host protein known to sequester metal ions is the antimicrobial protein calprotectin (Corbin et al., 2008), a heterodimer of the two EF-hand calcium-binding proteins S100A8 and S100A9 (Teigelkamp et al., 1991), which exerts antimicrobial activity against several bacterial and fungal organisms by sequestering zinc and manganese (Corbin et al., 2008; Urban et al., 2009; Hood et al., 2012; Liu et al., 2012). Upon dimerizing, S100A8 and S100A9 form two metal binding sites, both of which can bind strongly to Zn^2+^, though one is also capable of binding manganese (Kehl-Fie et al., 2011; Damo et al., 2013). Like lactoferrin and lipocalin-2, calprotectin is expressed by neutrophils, where it constitutes approximately 50% of their cytosolic content (Hessian et al., 1993). Calprotectin is thought to be secreted by apoptotic neutrophils, where it is associated with their extracellular traps, also called NETs (Urban et al., 2009). Similar to lipocalin-2, the two subunits of calprotectin, S100A8 and S100A9, are also induced by IL-17 and IL-22 in mucosal epithelial cells (Zheng et al., 2008; Liu et al., 2012; Zindl et al., 2013).

To successfully colonize the host, pathogens have evolved mechanisms to resist the effects of calprotectin-dependent zinc and manganese sequestration. Manganese is most notably important as a cofactor for enzymes that detoxify ROS (Aguirre and Culotta, 2012). Consistent with this role, manganese binding by neutrophil-derived calprotectin inhibits the growth of *S. aureus* in tissue abscesses and increases the susceptibility of this pathogen to oxidative stress (Kehl-Fie et al., 2011). To counteract this, the specialized manganese transporters MntABC and MntH contribute to systemic *S. aureus* infection by competing with calprotectin for manganese (Kehl-Fie et al., 2013). In *S. Typhimurium*, expression of the high-affinity zinc transporter ZnuABC aids the pathogen in overcoming calprotectin-mediated zinc sequestration and promotes the growth of *S. Typhimurium* in the inflamed gut as well as *Salmonella* competition with the microbiota (Liu et al., 2012). Genes encoding a similar ABC-type zinc transporter are present in *A. baumannii*, where they also mediate resistance to zinc sequestration by calprotectin and serve to increase pathogenesis (Hood et al., 2012).

S100A12 (calgranulin C) is another calgranulin protein like S100A8 (calgranulin A) and S100A9 (calgranulin B) which binds to zinc and other divalent cations. Similar to S100A8 and S100A9, S100A12 is also predominantly expressed by neutrophils, monocytes and activated macrophages (Robinson and Hogg, 2000). However, unlike S100A8 and S100A9, S100A12 is not found in rodents and its role in metal sequestration is not well-defined. S100A12 seems to be mainly pro-inflammatory through the activation of mast cells but may also play a role in chemotaxis (Hsu et al., 2009; Perera et al., 2010). S100A12 has antiparasitic activity against filarial nematodes (Gottsch et al., 1999), although this activity does not appear to be dependent on metal sequestration. Calcitermin, a 15-residue C-terminal cleavage fragment of S100A12, can be found in the human airways and exhibit antimicrobial activity against *E. coli, Pseudomonas aeruginosa*, and *C. albicans*, both at low pH and in media with zinc (Cole et al., 2001).

Another S100 protein with antimicrobial and immunomodulatory activity is S100A7, which is largely expressed in the skin and other epithelia. This protein, also known as psoriasin, was originally discovered as an abundant protein in psoriatic keratinocytes (Gläser et al., 2005). Psoriasin is secreted by keratinocytes and has antimicrobial activity against *E. coli*, possibly by sequestering zinc (Gläser et al., 2005). The molecule is considered an important effector molecule of the cutaneous barrier and, like S100A8 and S100A9, is also induced by IL-17 and IL-22 (Liang et al., 2006).

## Host vs. pathogens: the battle for metals at the intersection of health and disease in the mucosa

Sequestration of metal ions is one of the most important host strategies to limit the growth of bacterial and fungal pathogens. Metal limitation in the host is further enhanced during infection by the secretion of antimicrobial proteins that sequester metal ions, such as lipocalin-2 and calprotectin. Lipocalin-2 appears to be most effective in limiting the growth of commensal bacteria, as a number of pathogens have evolved or acquired additional siderophores to evade this response. In contrast, calprotectin restricts the growth of a variety of bacterial and fungal pathogens, including *S. aureus, C. albicans, B. burgdorferi, A. baumannii, and Aspergillus nidulans* (Lusitani et al., 2003; Urban et al., 2009; Moore et al., 2013). While both antimicrobial proteins are constitutively expressed by neutrophils, their expression—as well as the expression of other S100 proteins—may be induced in epithelial cells by pro-inflammatory stimuli like the Th17 cytokines IL-22 and IL-17 (Boniface et al., 2005; Zheng et al., 2008; Kerkhoff et al., 2012; Lee et al., 2012; Liu et al., 2012; Bando et al., 2013; Zindl et al., 2013).

**Figure 1:**
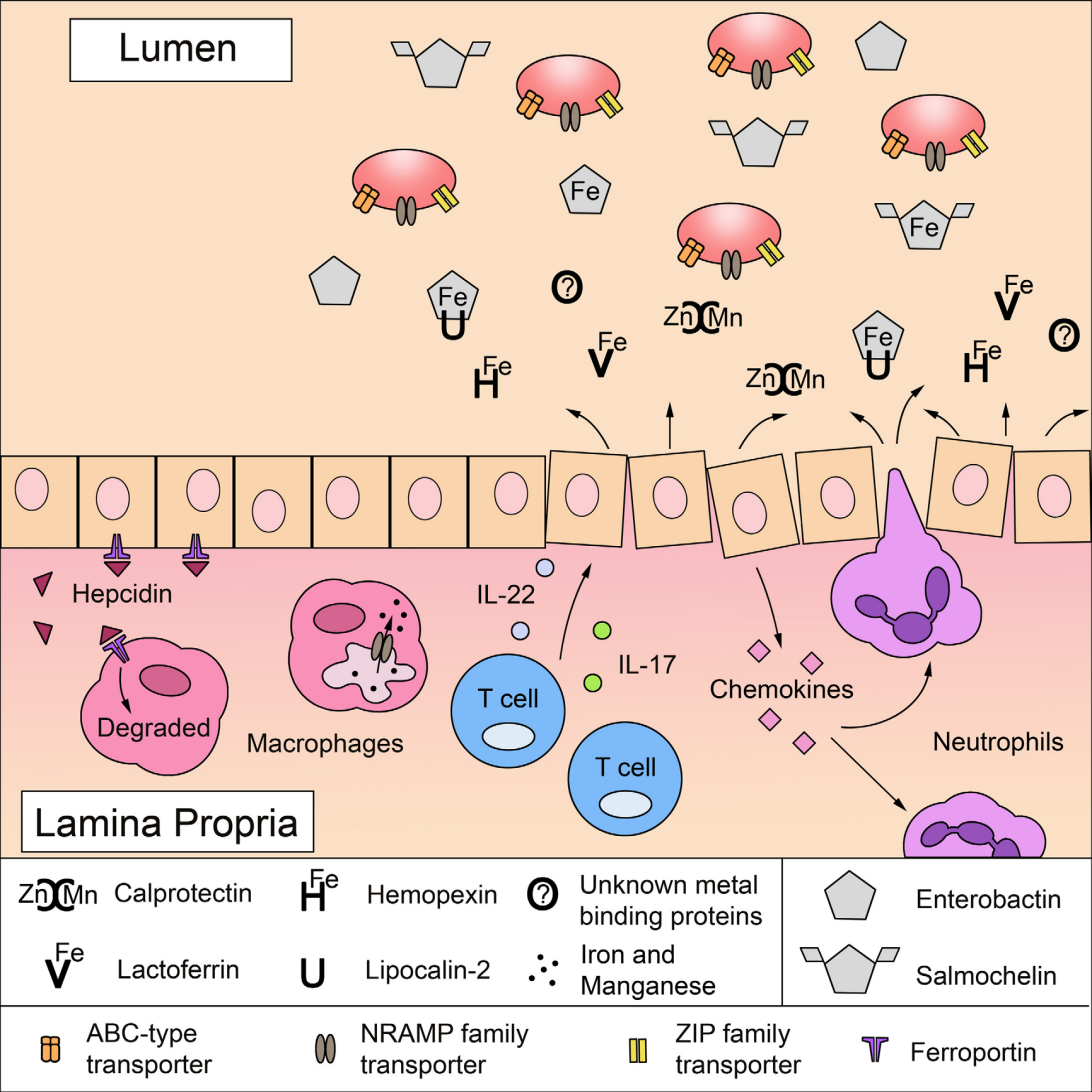
**A battle for metals in the intestinal mucosa: mechanisms of host metal sequestration and microbial metal acquisition**. To limit microbial growth, the mammalian host sequesters free iron, zinc, and manganese ions by expressing proteins in the mucosa that directly bind metals or metal-binding agents in a process termed nutritional immunity. Lactoferrin binds to iron (Fe) and calprotectin binds to zinc (Zn) and manganese (Mn). Hemopexin can limit the amount of circulating iron-bound heme (He) and lipocalin-2 sequesters the bacterial iron-scavenging siderophore enterobactin. Upon infection, inflammatory mediators increase the expression of metal-sequestering proteins, which is detrimental to microbes lacking mechanisms to survive metal deprivation. Inflammatory cytokines, IL-17 and IL-22, produced by T cells induce epithelial cells to express antimicrobial proteins including lipocalin-2 and calprotectin. Furthermore, activated epithelial cells secrete CXC chemokines that recruit neutrophils to the site of infection; neutrophils also express high levels of lactoferrin, lipocalin-2, and calprotectin. Microbial infection and inflammation can stimulate the production of hepcidin in the liver and in macrophages, which further reduces iron availability by inducing the degradation of the cellular iron exporter ferroportin 1. In addition, the divalent metal ion transporter NRAMP1 can export manganese and iron out of the macrophage phagosome to further restrict metal availability to intracellular pathogens. To overcome metal starvation, pathogens (red ovals) employ several strategies to acquire iron, zinc and manganese. Highly specialized ABC-type transporters facilitate the uptake of zinc and manganese as well as iron bound to heme and siderophores. Siderophores, such as enterobactin, are iron-scavenging agents. Although lipocalin-2 can sequester enterobactin to limit microbial access to iron, some pathogens use salmochelin, a C-glucosylated derivative of enterobactin that cannot be bound by lipocalin-2. Some pathogens also express NRAMP family transporters and ZIP family transporters for the uptake of manganese and zinc.

Altogether, the secretion of antimicrobial proteins and the production of reactive oxygen and nitrogen species at the site of infection can reduce the growth of many microorganisms. Susceptibility of commensal bacteria to ROS may be exacerbated as a result of lipocalin-2 and calprotectin expression because these proteins sequester metals that serve as cofactors in bacterial enzymes responsible for neutralizing free radical species. However, in the harsh environment these responses create, microbes with metal scavenging ability can often survive and replicate, sometimes even dominating when commensal competition is reduced. This is the case for *S. Typhimurium*, which overcomes both lipocalin-2- and calprotectin-mediated metal sequestration to colonize the inflamed gut and compete with the microbiota (Raffatellu et al., 2009; Liu et al., 2012), a theme that likely applies to other pathogens (Figure 1). Therefore, the secretion of antimicrobial proteins like lipocalin-2 and calprotectin may have a detrimental effect on the host by killing commensal bacteria that are more susceptible to oxidative damage, neutrophil enzymatic activity, and metal nutrient deprivation. Elevated lipocalin-2 and calprotectin levels observed in patients with inflammatory bowel disease (Cayatte et al., 2012; Østvik et al., 2013; Wang et al., 2013) may also be detrimental to the host because antimicrobial activity toward commensal bacteria likely contributes to the microbial imbalance observed in the digestive tract of these patients, known as dysbiosis (Salzman and Bevins, 2008; Manichanh et al., 2012). Sustained intestinal dysbiosis can lead to the overgrowth of potentially harmful bacteria termed pathobionts (Stecher et al., 2013).

The fact that transition metals are essential for proper development and function of the host further complicates the host metal economy during infection. For example, zinc is needed for immune development and function, but it also has to be sequestered from staphylococcal abscesses in order to zinc-starve *S*. *aureus* during infection (Kehl-Fie et al., 2013). Furthermore, the amounts of metals vary in different organs (Kehl-Fie et al., 2013), which may be a basis for site-specific differences in the host's metal sequestration strategies. A contributing factor in these differences may include microbial colonization. In the healthy gut, and possibly at other mucosal sites colonized by commensal bacteria, host interactions with the microbiota likely regulate the low-level expression of metal binding proteins as well as the concentration of transition metals. At these mucosal sites and in other tissues and organs, it is plausible that other antimicrobial proteins besides lipocalin-2 and calprotectin may sequester metal ions but have yet to be identified.

In concert with other host defense strategies, nutritional immune responses at the mucosa can lead to beneficial outcomes for the host by reducing the colonization of invading pathogens. However, they can also alter the normal microbial flora, which may enhance the colonization of pathogens like *S. Typhimurium* or result in dysbiosis. Thus, it is important to take into account that metal sequestration strategies can be beneficial to the host, but may also potentially benefit pathogens or pathobionts that evade these responses. Moreover, investigating the mechanisms of host-microbe competition for metal ions may pave the way for developing novel therapeutics that are in critical need given the mounting global threat of antibiotic-resistant pathogens and pathobionts.

### Conflict of interest statement

The authors declare that the research was conducted in the absence of any commercial or financial relationships that could be construed as a potential conflict of interest.
